# Natural regeneration on seismic lines influences movement behaviour of wolves and grizzly bears

**DOI:** 10.1371/journal.pone.0195480

**Published:** 2018-04-16

**Authors:** Laura Finnegan, Karine E. Pigeon, Jerome Cranston, Mark Hebblewhite, Marco Musiani, Lalenia Neufeld, Fiona Schmiegelow, Julie Duval, Gordon B. Stenhouse

**Affiliations:** 1 Caribou Program, fRI Research, Hinton, Alberta, Canada; 2 Arctos Ecological Consultants, Edmonton, Alberta, Canada; 3 Wildlife Biology Program, Department of Ecosystem and Conservation Science, W.A. Franke College of Forestry and Conservation, University of Montana, Missoula, Montana, United States of America; 4 Department of Biological Sciences, Faculty of Science, University of Calgary, Calgary, Alberta, Canada; 5 Parks Canada, Jasper National Park, Jasper, Alberta, Canada; 6 Department of Renewable Resources, University of Alberta, Edmonton, Alberta, Canada; 7 GIS Program, fRI Research, Hinton, Alberta, Canada; 8 Grizzly Bear Program, fRI Research, Hinton, Alberta, Canada; Université de Sherbrooke, CANADA

## Abstract

Across the boreal forest of Canada, habitat disturbance is the ultimate cause of caribou (*Rangifer tarandus caribou*) declines. Habitat restoration is a focus of caribou recovery efforts, with a goal to finding ways to reduce predator use of disturbances, and caribou-predator encounters. One of the most pervasive disturbances within caribou ranges in Alberta, Canada are seismic lines cleared for energy exploration. Seismic lines facilitate predator movement, and although vegetation on some seismic lines is regenerating, it remains unknown whether vegetation regrowth is sufficient to alter predator response. We used Light Detection and Ranging (LiDAR) data, and GPS locations, to understand how vegetation and other attributes of seismic lines influence movements of two predators, wolves (*Canis lupus*) and grizzly bears (*Ursus arctos*). During winter, wolves moved towards seismic lines regardless of vegetation height, while during spring wolves moved towards seismic lines with higher vegetation. During summer, wolves moved towards seismic lines with lower vegetation and also moved faster near seismic lines with vegetation <0.7 m. Seismic lines with lower vegetation height were preferred by grizzly bears during spring and summer, but there was no relationship between vegetation height and grizzly bear movement rates. These results suggest that wolves use seismic lines for travel during summer, but during winter wolf movements relative to seismic lines could be influenced by factors additional to movement efficiency; potentially enhanced access to areas frequented by ungulate prey. Grizzly bears may be using seismic lines for movement, but could also be using seismic lines as a source of vegetative food or ungulate prey. To reduce wolf movement rate, restoration could focus on seismic lines with vegetation <1 m in height. However our results revealed that seismic lines continue to influence wolf movement behaviour decades after they were built, and even at later stages of regeneration. Therefore it remains unknown at what stage of natural regeneration, if any, wolves cease to respond to seismic lines. To reduce wolf response to seismic lines, active restoration tactics like blocking seismic lines and tree planting, along with management of alternate prey, could be evaluated.

## Introduction

Habitat disturbance and loss are recognised as key factors in the loss of global biodiversity [1,2]. Anthropogenic habitat disturbance can reduce the accessibility of natural resources for wildlife [1], directly and indirectly increase wildlife mortalities [3], and also have more subtle effects such as long-term reduction of habitat quality and function [4], and altered predator-prey dynamics [5]. However, not all anthropogenic disturbances are equal. In addition to environmental and biological factors that affect individual responses (e.g. reproductive status, season, and weather), wildlife can respond differently to disturbances with respect to the activity severity of the disturbance [6], and time since disturbance [7]. In areas where anthropogenic disturbances are years, or even decades old, natural regeneration (shrubs, grasses, and trees) may negate the need for active restoration, although it is unclear whether regenerative vegetation is sufficient to change wildlife responses. Given the widespread alteration of landscapes by humans, and the limited resources available, a triage approach may be required to direct restoration and management to areas that will have the most benefit for wildlife [8]. By identifying when regenerated areas no longer negatively impact wildlife species that are of conservation concern, we can direct mitigation activities where they are most needed.

The boreal forest of western Canada is fragmented by an extensive footprint of anthropogenic disturbance in the form of forest clear cuts, well sites, pipelines, power lines, seismic lines, and access roads [9,10]. The negative effects of habitat fragmentation on boreal wildlife are well documented [11,12], and of particular concern are boreal and central mountain woodland caribou (*Rangifer tarandus caribou*). Once widespread throughout the boreal forest and mountains of western Canada respectively, boreal and central mountain woodland caribou have declined across their range [13,14]. Ultimately, these declines are believed to be caused by habitat disturbances associated with land use and management activities [13,15]. Large areal disturbances like forestry, oil and gas, and mining activities have resulted in numerical responses by ungulates that prefer early seral forest (e.g. moose (*Alces alces*), deer (*Odocoileus* spp.), and elk (*Cervus canadensis*)), and correspondingly increased the numerical response of shared predators such as wolves (*Canis lupus*) within caribou ranges [16–18]. Linear disturbances (roads, pipelines, and seismic lines) also increase functional overlap between these shared predators and caribou [19,20]. As a result, cumulative anthropogenic disturbance has increased apparent competition between caribou and ungulates that prefer early seral stages via increased predation [21,22].

Recognizing the negative effects of anthropogenic disturbances on the persistence of caribou, the Canadian federal government recovery strategies for boreal and southern mountain caribou state that at least 65% of the range of each caribou herd (low elevation winter and connectivity range for central mountain caribou) should be undisturbed [10,23]. In Alberta Canada, however, all caribou ranges currently exceed this threshold, populations are declining at an average of 8% per year [14,24], and there is an urgent need to implement habitat restoration as a measure to facilitate caribou recovery. Legacy seismic lines (linear features approximately 8 m wide that were cleared throughout the boreal forest during seismic soundwave mapping of oil reserves prior to 1990, hereafter seismic lines) have been a focus of scientific inquiry because they increase functional overlap between predators and caribou by facilitating predator movement and increasing caribou and predator encounters [20,25–27]. Specifically, predator movement rates on seismic lines can be up to double those in the forest interior [25], and even low densities of seismic lines increase predator use of habitats [28,29].

The extensive seismic line footprint within Alberta means that restoration will need to be prioritized [8]. Until recently, quantification of seismic line regeneration was not feasible at a large scale and corresponding investigations of wildlife response to regeneration were rare (but see [28,30,31]). Light Detection and Ranging Data (LiDAR) now facilitates detailed and broad scale assessments of habitat regeneration in three dimensional space, including measuring vegetation height on seismic lines [32]. We used animal Global Positioning System (GPS) data and LiDAR-based measurements of vegetation height on seismic lines to assess movement ecology of wolves and grizzly bears in relation to regenerating seismic lines. Our goals were 1) to determine whether natural regeneration on seismic lines is sufficient to make predator use of seismic lines indistinguishable from the surrounding landscape, and in the context of habitat restoration priorities for caribou, 2) to understand what specific characteristics of seismic lines make seismic lines most attractive to predators for movement.

At a broad scale, we assessed movement behaviour using step selection functions [33]. We predicted that at this broad scale, grizzly bears and wolves would move towards seismic lines that are attractive movement routes. Specifically that both species would move towards seismic lines i) with lower vegetation height, as seismic lines with lower vegetation height are more attractive movement routes when compared to seismic lines with higher vegetation height [30], ii) with dry soil, as seismic lines with dry soil likely facilitate faster movement rates than seismic lines with wet soil, and also likely have less vegetative cover to impede movement, iii) that fall within forest, as seismic lines that fall in forest are likely more attractive as movement routes than seismic lines in non-forest, and iv) that fall within areas with lower densities of seismic lines, as seismic lines that occur in areas where there are fewer seismic lines are likely more attractive as movement routes than seismic lines that fall within areas with more seismic lines. At a fine scale, as lower vegetation on seismic lines facilitates faster predator movement rates, we predicted that movement rates near seismic lines would increase with decreasing vegetation height of seismic lines [30]. Detailed predictions and associated models are described in Table 1. Improving our understanding of how regeneration of disturbed areas affects movement ecology of two predators will help determine when regenerated disturbances no longer impact wildlife of conservation concern. In addition, by evaluating predator movement behaviour relative to regenerating seismic lines our research could be used to aid in recovery and conservation efforts for caribou.

**Table 1:** Models and associated predictions used to explain movement behaviour and movement rates of wolves and grizzly bears in relation to seismic lines in west-central Alberta, Canada, between 2003 and 2009.

Model (M)	Prediction
**Step Selection Functions**	
M1.eDist	Null model
M2.eDist*Veght	A. Move towards (eDist) lower vegetation height (Veght) seismic lines.
M3.eDist*Veght*eWAM	B. Move towards (eDist) drier (eWAM), lower vegetation height (Veght) seismic lines.
M4.eDist*Veght* Density	C. Move towards (eDist) lower vegetation height (Veght) seismic lines in areas with lower densities of seismic lines (Density).
M5.eDist*Veght*fLand	E. Move towards (eDist) lower vegetation height (Veght) seismic lines in forest (fLand(Con), fLand(Mix)).
**Movement Rate**	
M6.Season	Null Model
M7.Veght*Season	G. Increased movement rate near lower vegetation height (Veght) seismic lines.
M8.Veght*Season*fFor	H. Increased movement rate near lower vegetation height (Veght) seismic lines in forest (fFor(1)).

Variables are further described in S1 Table.

## Materials and methods

### Ethical statement

Animal capture and handling protocols adhered to guidelines under the Canadian Council on Animal Care [34] and were approved by university animal care committees (University of Alberta Animal Care Committee Standards 99–69; University of Calgary Animal Use Protocol BI11R-17; University of Montana Animal Use Protocol 059-09MHWB-122209; University of Saskatchewan Animal Use Protocol 20010016) and the Alberta Department of Sustainable Resource Development Animal Care Committee. Capture occurred on public lands and in provincial parks, and permission for capture of animals was granted under the authority of the Government of Alberta.

### Study area

**Fig 1:**
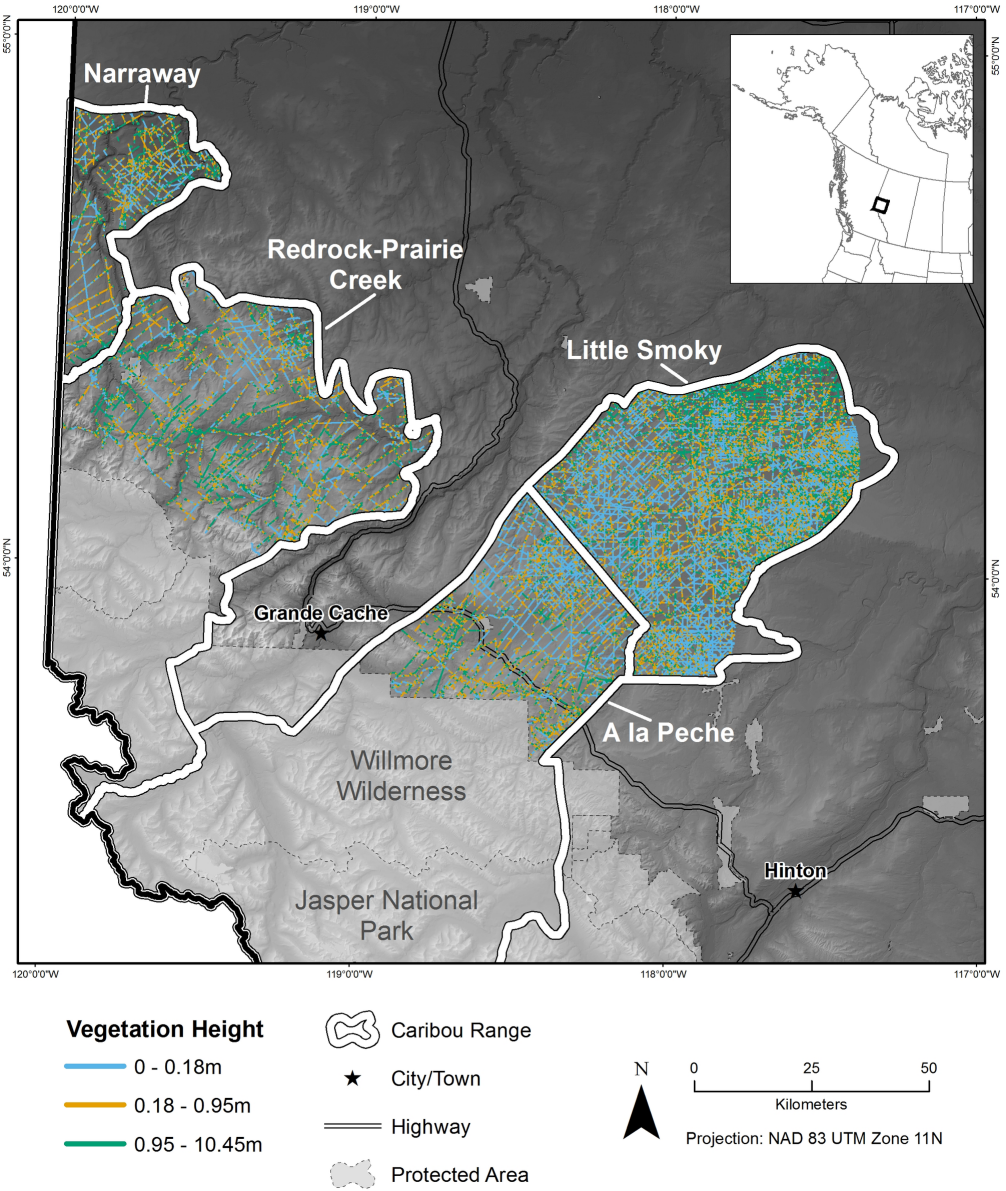
Vegetation height on seismic lines in west-central Alberta, Canada. Legacy seismic line footprint (15,588 km) within the range of west-central Alberta, Canada, caribou herds attributed with vegetation heights (33%, 66% and 100% quantiles) using LiDAR.

The study area was in west-central Alberta, Canada (Fig 1; -117°W to -120°W, 53°N to 55°N) and encompassed the entire range of one boreal woodland caribou herd (Little Smoky), the low elevation winter ranges of three central mountain woodland caribou herds (A La Peche, Narraway, and Redrock-Prairie Creek), one grizzly bear population unit (BMA 4 Grande Cache), and seven wolf packs (A La Peche, Berland, Kakwa, Muskeg, Narraway, Simonette, and Two Lakes). We did not consider areas in the mountainous portions of central mountain caribou ranges because these areas are largely within protected areas with low human footprint. The study area was 10,772 km^2^ and spanned two natural sub-regions (upper foothills and lower foothills [35]). There are 15,588 km of seismic lines within the study area and other industrial footprint includes forestry cut blocks, oil and gas pipelines, access roads, and well sites. Mean seismic line densities were 1.45 km/km^2^.

### Animal location data

The animal GPS-telemetry dataset consisted of multiyear wolf and grizzly bear locations. Between 2003 and 2004 wolves were captured using rubber-padded foothold traps and via aerial net-gunning [36], while between 2007 and 2009 wolves were captured using rubber-padded foothold traps [37]. Grizzly bears were captured using leg-hold snares, culvert traps, and aerial helicopter darting [38,39], with capture efforts focused on culvert traps and aerial darting from 2006 onwards [40].

For wolves, data were collected from 24 individuals (17 female, 7 male) from 7 packs (A La Peche n = 2, Berland n = 2, Kakwa n = 4, Muskeg n = 7, Narraway n = 1, Simonette n = 5, Two Lakes n = 2) between 2003 and 2007, or 2007 to 2009 inclusively. We rarefied GPS locations to 2-hour intervals (Lotek 2200/3300, Lotek Engineering Systems, Newmarket, Ontario, Canada or Televilt GPS Simplex, Lindesberg, Sweden). For grizzly bears, we used GPS locations rarefied to 1-hour intervals (Televilt Global Positioning System, Lindesberg, Sweden) collected between 2005 and 2009 from 19 individuals (9 male, 10 female). Because of divergent habitat selection patterns among reproductive status and sex, we divided grizzly bear data into male and female groups, and further partitioned adult females into those with cubs <1 year old, and those without cubs and with cubs >1 year old (female [7]). The presence of cubs was confirmed visually during telemetry flights or capture events, and females with no cub observed during at least two repeated observations were classified as lone females. We excluded females with cubs <1 year old from analyses because of small sample sizes (n = 3, fall season only). We did not partition wolf data into sex or reproductive status because patterns of resource selection are similar for males and females [41].

In addition, we partitioned data based on seasonal resource availability patterns of grizzly bears (spring 1 May to 15 June; summer 16 June to 31 July; fall 1 August to 15 October [42]) and wolves (denning 20 April to 30 June; rendezvous 1 July to 20 September; nomadic 21 September to 19 April [36]). Because we were using GPS locations from earlier-technology collars with low fix success rate (wolves mean 0.51 (range 0.24–0.79); grizzly bears mean 0.46 (range 0.28–0.72)), we weighted all locations with the inverse of the probability of obtaining a fix (PFix) using models developed by Frair et al. [43] and Hebblewhite et al. [44].

### Step selection functions–broad scale movements

Because linear features like seismic lines influence predator movement, we confined our analysis to GPS locations collected when animals were actively moving, rather than resting or feeding. We used a clustering tool developed using the ArcPy site package within ArcGIS 10.2.2 [45] to divide GPS locations into ‘movement’ and ‘stationary’ (when animals were resting or feeding on a kill). For wolves, we defined stationary locations as locations collected when animals spent more than 6 hours within a 300-m radius [46]. We defined grizzly bear stationary locations as locations collected when bears spent more than 7 hours within a 100-m radius to exclude bedding and kill sites, but include foraging and travel. We excluded stationary locations for both species and also excluded animals with less than 40 movement locations per season from the analysis dataset. The step selection function dataset included 4,667 wolf locations and 9,658 grizzly bear locations (S2 Table).

### Movement rates–fine scale movements

The relatively low number of GPS locations available for analysis in the study area, and associated location error of ± 30 m in forest stands for the earlier-technology collars we used [47] prevented us from assessing movement along seismic lines that are on average, only 8 m wide as reported by Dickie et al. [25,30]. Thus, to balance potential GPS measurement error while ensuring that we had a sufficient number of locations to model animal movement near seismic lines across all seasons, we used GPS locations from animals with at least 20 locations that fell within 100 m of seismic lines within each season, reflecting locations that were well within the zone of influence of movement of seismic lines [48]. We recognize that the fix-interval available for analysis (1 and 2 hour) may underestimate animal movement rates [49,50], however within our study area fine scale data (e.g. 5 min fixes) were not available that matched the time that the LiDAR were collected.

Previous research assessing wolf movement rates revealed significant differences between males and females [51], however we had insufficient data (1 male) to build male-specific seasonal models or to include sex as a factor within models, we therefore only included female wolves in the movement rate dataset, the grizzly bear movement rate dataset included males and females. The movement rate dataset included 813 wolf locations (17% of movement locations) and 2,973 grizzly bear locations (31% of movement locations; S2 Table).

### Data analysis

Prior to analyses, we screened data for non-linearity, collinearity, and correlations following Zuur et al. [52], and excluded one of two variables from the same model if correlation coefficients were greater than 0.6, or if variation inflation factors were greater than 3. We carried out data exploration and statistical analyses within R [53] and visualised results using the ggplot2 package [54].

### Step selection functions–broad scale movements

We used step selection functions to assess the effect of seismic line regeneration, landcover, and seismic line wetness on movement of wolves and grizzly bears. Step selection functions (SSF [33]) integrate analysis of movement in a used-available resource selection analysis framework, providing a movement-based definition of availability and improving the definition of availability from an explicit movement-modeling paradigm [55]. We used Geospatial Modelling Environment [56] and ArcGIS 10.2.2 [45] to summarise movement distances and turn angles between successive GPS locations, and tested for correlations between movement distance and turn angles of each used step for each species-sex group within each season. We found no significant correlations (*r*_*s*_ <0.115 in all cases) and therefore generated 10 available steps for each used step by randomly drawing step lengths and turn angles from the observed movement distributions for each species-sex group within each season [57].

We analysed data using conditional logistic regression [58] in the survival package [59], with each stratum consisting of one used step and 10 available steps. For wolves, we controlled for correlated movements of pack members by randomly retaining only one step when wolves from the same pack travelled within 200 m of each other [60,61]. Also for both species, as successive steps by an individual can be correlated with one another [55], we calculated robust standard errors based on independent clusters of steps following Fortin et al. [33]. We considered wolf steps that occurred more than five days apart from one another as independent clusters [61], while grizzly bear steps were considered independent from one another after 24 hours [62]. This approach yielded 67 independent wolf clusters and 307 independent grizzly bear clusters (S2 Table).

We fit conditional logistic regression models using a general estimating equation [59], and thus used the quasi-likelihood under the independence model criterion (QIC_U_ [63]) to assess which model(s) (Table 1) best explained observed animal steps (MuMIn package [64]). Because we were interested in typical selection patterns within the study area, we carried out QIC_U_ model selection at the population level [65], and chose the best model based on the lowest population QIC_U_ and highest model weight (*ω*_*i*_).

To calculate population-level coefficients, we fit the best model to each individual and then inverse-weighted coefficients across individuals [66]. We report results as beta (β) coefficients ± 95% confidence intervals (95% CI), and as the relative probability (logit (P)) of step selection as a function of exp ^(β1 +… βx)^/(1+exp^(β1 +… βx)^).

We evaluated models with k-fold cross validation [67] using the hab package [68]. We randomly partitioned our strata into 80% training data and 20% testing data, and calculated the correlation (r_s_) between the relative probabilities of observed and predicted data for each used and available step. We repeated the process 100 times and report the average and range of r_s_ values for used (r_s1_) and available (r_s0_) steps across all 100 comparisons; with better model performance indicated by higher values of r_s1_ when compared to r_s0_.

### Movement rates–fine scale movements

We used linear mixed models within the lme4 package [69] to assess movement rates of wolves and grizzly bears in relation to vegetation height and habitat intersecting seismic lines (S1 Table). Movement rates were exponentially distributed for both species, we therefore log_e_ transformed movement rate and modeled movement rate as a Gaussian distribution. For wolves, we built a single model that included season as an interaction and identified the best random effect structure for the model (individual, individual nested within pack) using Akaike’s Information Criterion corrected for small sample sizes (AIC_C_ [70]). To ease interpretation, we divided distances travelled by wolves between consecutive 2 hour locations by a value of 2, therefore converting the units to m/hour. For grizzly bears we built models for each sex and included season as an interaction. Vegetation height data were Poisson distributed, we therefore log_e_ transformed the vegetation height variable (*Veght*) after adding a constant of 1 to meet the linearity assumption for linear predictors.

We identified which model (Table 1) best explained movement rates using AIC_C_ calculated in the AICcmodavg package [71]. We assessed the fit of the best model using marginal (R^2^_LMM(m)_) and conditional R^2^ (R^2^_LMM(c)_) values for linear mixed models calculated using the MuMIn package [64], where R^2^_LMM(m/c)_ is the proportion of variance explained by the fixed effects, and combined random and fixed effects, within the model respectively [72].

When the best model revealed that there was a relationship between vegetation height and movement rate, we used piecewise regression to identify breakpoints where the relationship between vegetation height, and movement rate for each species, sex, and season changed. We carried out piecewise regression with the SiZer package [73] and estimated confidence intervals using 1,000 bootstrap replicates with α set at 0.05.

### Environmental data

Our motivation was to assess the overall influence of seismic lines on animal movement, therefore, we only included distance to seismic lines and environmental attributes of seismic lines within our analysis. Detailed SSF-based habitat models for wolves and grizzly bears can be found elsewhere [74,75].

### Attributes of seismic lines

We used LiDAR data collected between 2005 and 2007 during the leaf-on period to attribute vegetation height to 15,588 km of seismic lines within the study area (Fig 1); calculating mean vegetation height (variable names in italics: *Veght*) along approximately 100 m length segments of seismic lines using a least-cost approach which identified and quantified the lowest vegetation along the seismic line (i.e. game trails; S1 File). We measured seismic line wetness, as represented by mean depth to water of each seismic line segment, using wet areas mapping data (WAM [76]). To represent the diminishing effect of the depth to water on vegetation growth we transformed WAM using an exponential decay function (*eWAM*: 1-exp^-1.55*WAM(m)^) that caused the effect of depth to water to rapidly decrease at depths greater than 2 m, and to become constant at depths greater than 3 m (mean root depth of boreal forest vegetation 2 ± 0.3 m [77]).

Using landcover derived from Moderate Resolution Imaging Spectroradiometer (MODIS) and Landsat imagery mapped at a 30 m x 30 m resolution [78], we determined the landcover (*fLand*) that intersected the longest length of each 100 m seismic line segment. For step selection function analysis, we classified landcover into three categories: Conifer (*fLand*:*Con*), Mixed (*fLand*:*Mix*), and Non-forest (*fLand*:*NF*). For movement rate analysis we classified landcover into a binary variable (*fFor*: 1 Forest, 0 Non-forest). For step selection function analyses, we also used a 1 km moving window with a 30 m cell size to calculate the density (*Density*) of seismic lines across our study area (km/km^2^). We attributed 100 m seismic line segments with mean *Veght*, *eWAM*, and *Density* values, and with the landcover (*fLand*, *fFor*) that intersected the majority of the seismic section using Geospatial Modelling Environment [56] and ArcGIS 10.2.2 [45,57]. Attributes of seismic lines are described in S1 Table.

### Attributes of animal steps

For step-selection function analyses, to represent the diminishing effect of seismic lines and attributes of seismic lines on movement behaviour with increasing distance to seismic lines, we used an exponential decay function as the measure of distance from the end of each step to each seismic line segment (*eDist*; 1-exp^-0.002*Distance(m)^; S1 Table). As outlined by Thurfjell [57] because linear features are narrower than the step length of the animal only a small portion of each step will contain the linear feature, so using metrics calculated along the step might underestimate selection. Therefore, we used the end of the step to avoid underestimating selection for narrow seismic lines [57]. The exponential decay function caused the effect of distance to decrease rapidly beyond 500 m, and to become constant at distances greater than 2 km [42].

## Results

### Vegetation height

Mean vegetation height along seismic lines within the study area was 0.81 m (range 0–15 m; standard deviation 1.3 m; Fig 1). 11,755 km (75%) of the seismic line footprint had a mean vegetation height less than 1 m (S1 Fig).

### Step selection functions–broad scale movements

#### Wolves

During the denning season, the model including an interaction between distance to the nearest seismic line (*eDist*), seismic line vegetation height (*Veght*), and landcover intersecting the seismic line (*fLand*) best explained wolf steps (M5, ω_i_ = 1). During the rendezvous season the model including an interaction between distance to the nearest seismic line, seismic line vegetation height, and seismic line wetness (*eWAM*) best explained wolf steps (M3, ω_i_ = 0.85), and during the nomadic season the model including distance to the nearest seismic line best explained wolf steps (M1, ω_i_ = 0.99; Table A in S2 File). Averaged inverse-weighted coefficients for the best models for each season are in Table A in S3 File.

**Fig 2:**
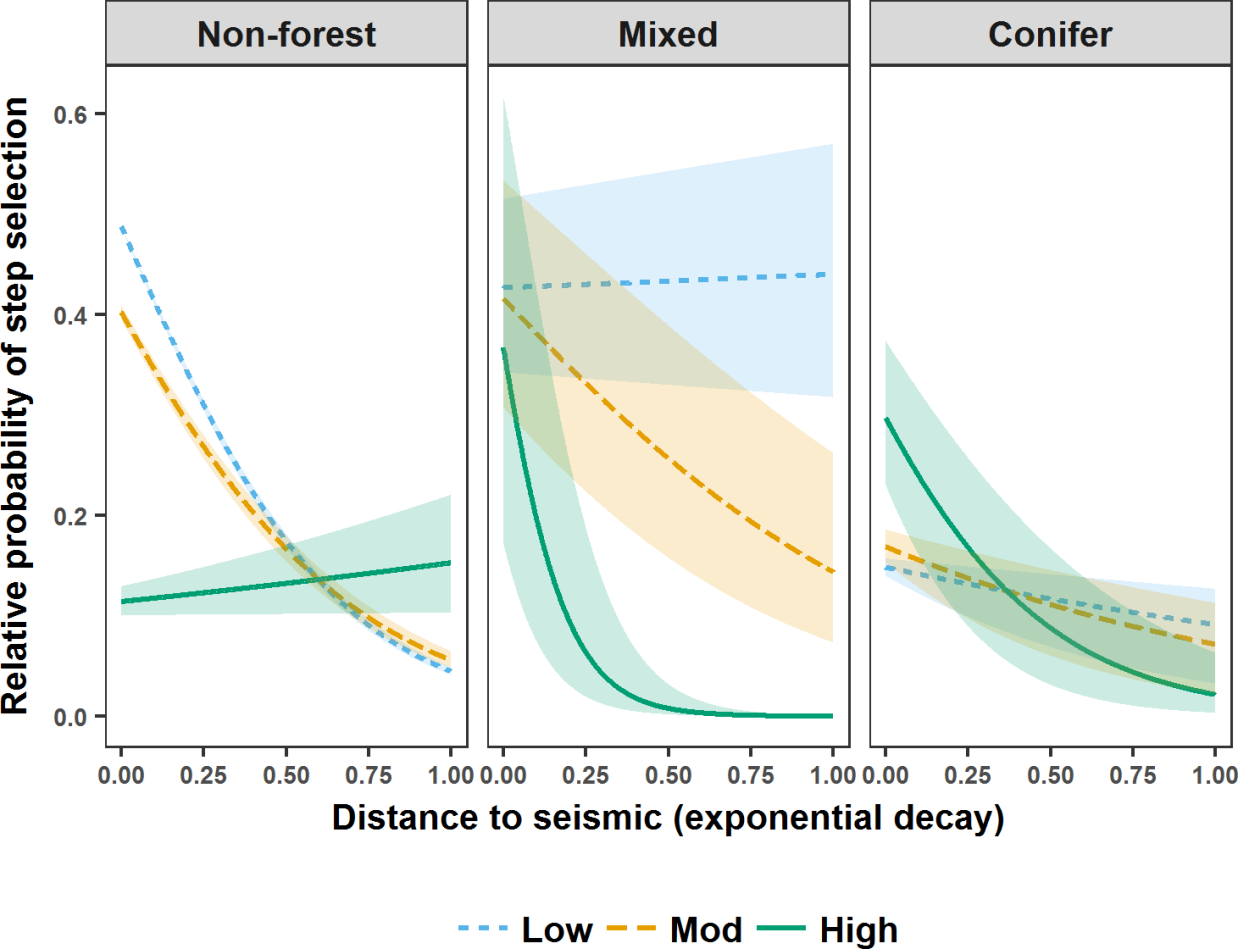
Relative probability of step selection by wolves in west-central Alberta during the denning season. Relative probability of step selection by wolves in west-central Alberta between 2003 and 2009 during the denning season in relation to distance to seismic lines (represented as an exponential decay (1-exp^-0.002* Distance (m)^)), seismic line vegetation height (visualised using the mean of the lower (Low), middle (Mod), and upper (High) quantiles), and landcover intersecting seismic lines (Non-forest, Mixed, Conifer). Shaded areas are 95% confidence intervals around relative predicted probabilities of step selection.

**Fig 3:**
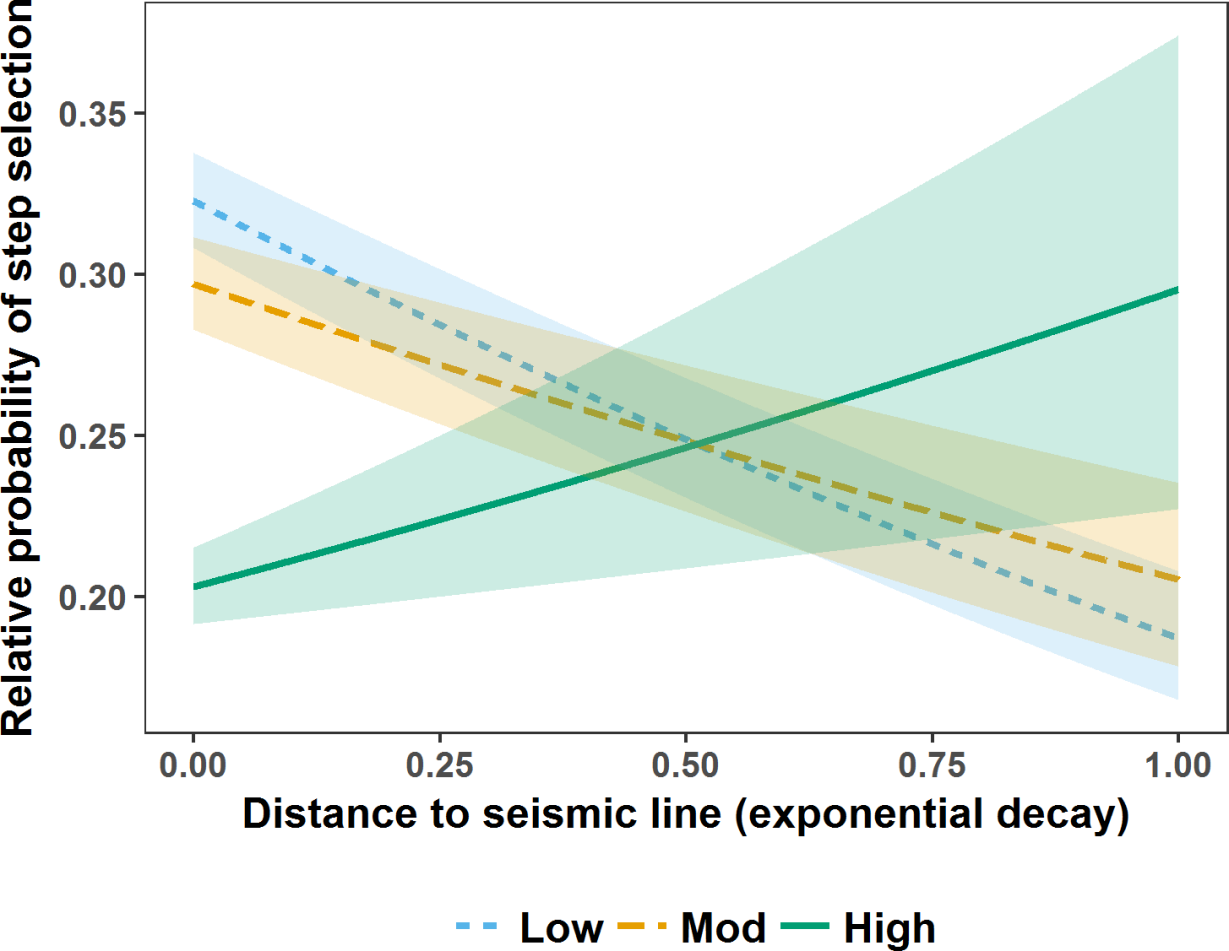
Relative probability of step selection by wolves in west-central Alberta during the rendezvous season. Relative probability of step selection by wolves in west-central Alberta between 2003 and 2009 during the rendezvous season in relation to distance to seismic lines (represented as an exponential decay (1-exp^-0.002* Distance (m)^)) and seismic line vegetation height (visualised using the mean of the lower (Low), middle (Mod), and upper (High) quantiles). Seismic line wetness (*eWAM*) was held at the mean for prediction. Shaded areas are 95% confidence intervals around relative predicted probabilities of step selection.

During the denning season M5 indicated that wolf steps brought them closer to seismic lines (Fig 2). Specifically, wolf steps brought them closer to lower vegetation height seismic lines in non-forest, and closer to higher vegetation height seismic lines in mixed and conifer forest (Fig 2). During the rendezvous season M3 indicated that regardless of seismic line wetness, wolf steps brought them closer to lower vegetation height seismic lines (Fig 3). During the nomadic season M1 indicated that wolf steps brought them closer to seismic lines (β_*eDist*_ = -0.33, 95% CI = 0.01). Means and ranges of Spearman rank correlations from k-fold cross validation suggested that M5 accurately predicted wolf steps during the denning season (mean r_s1_ 0.806, range 0.414–0.975; mean r_s0_ 0.015, range -0.695–0.709), but that M3 and M1 failed to completely predict wolf steps during the rendezvous (mean r_s1_ 0.559, range -0.253–0.924; mean r_s0_ 0.002, range -0.765–0.914) and nomadic (mean r_s1_ 0.226, range -0.314–0.799; mean r_s0_−0.003, range -0.887–0.768) seasons.

#### Grizzly bears

The model including an interaction between distance to the nearest seismic line (*eDist*), seismic line vegetation height (*Veght*), and landcover intersecting the seismic line (*fLand*) best explained female grizzly bear steps across all seasons, and also best explained male grizzly bear steps during fall (M5, ω_i_ = 1). During spring, the models including an interaction between distance to the nearest seismic line, seismic line vegetation height, seismic line wetness (*eWAM*), and landcover intersecting the seismic line best explained male grizzly bear steps (M3, ω_i_ = 0.65; M5, ω_i_ = 0.33). During summer, the model including distance to the nearest seismic line best explained male grizzly bear steps (M1, ω_i_ = 0.68; Tables B and C in S2 File). Averaged inverse-weighted coefficients for the best models for each season are in Tables B and C in S3 File.

**Fig 4:**
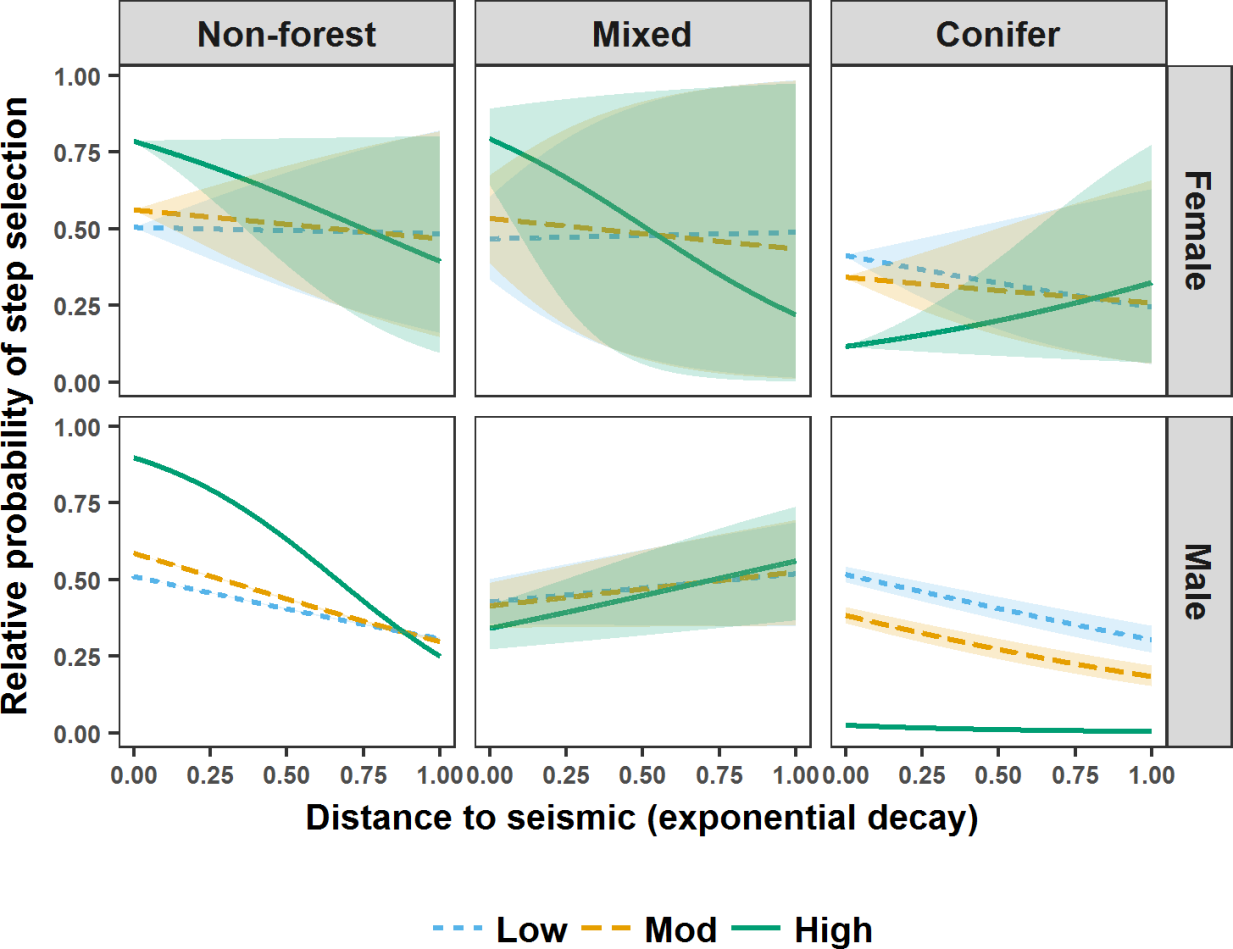
Relative probability of step selection by grizzly bears in west-central Alberta during spring. Relative probability of step selection by grizzly bears in west-central Alberta between 2005 and 2009 during spring in relation to distance to seismic lines (represented as an exponential decay (1-exp^-0.002* Distance (m)^)), seismic line vegetation height (visualised using the mean of the lower (Low), middle (Mod), and upper (High) quantiles), and landcover intersecting seismic lines (Non-forest, Mixed, Conifer). Shaded areas are 95% confidence intervals around relative predicted probabilities of step selection.

**Fig 5:**
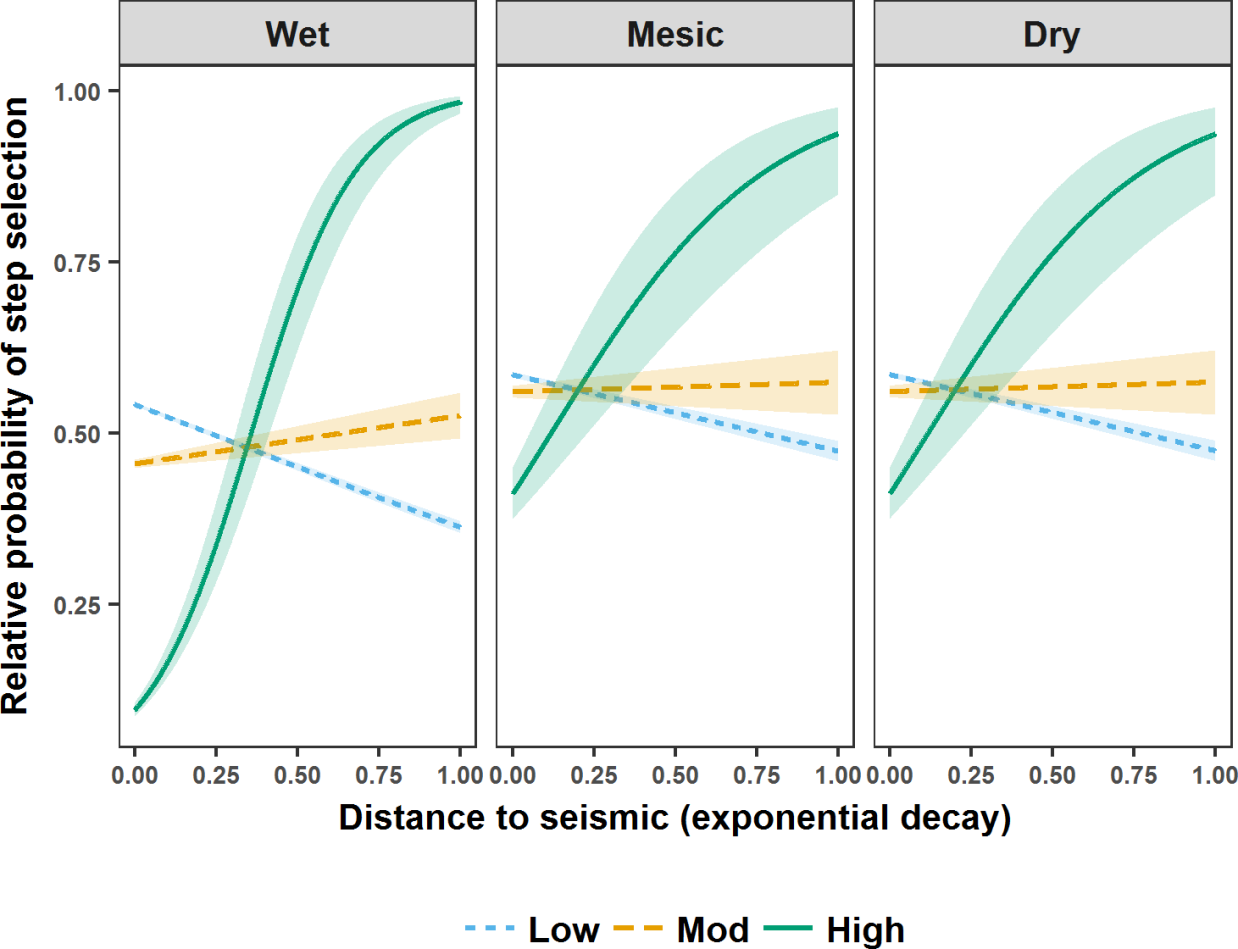
Relative probability of step selection by male grizzly bears in west-central Alberta during spring. Relative probability of step selection by male grizzly bears in west-central Alberta between 2005 and 2009 during spring in relation to distance to seismic lines (represented as an exponential decay (1-exp^-0.002* Distance (m)^)), seismic line vegetation height (visualised using the mean of the lower (Low), middle (Mod), and upper (High) quantiles), and seismic lines wetness (represented as an exponential decay (1-exp-1.55*WAM(m)) and visualised using the mean of the lower (Wet), middle (Mesic), and upper (Dry) quantiles. Shaded areas are 95% confidence intervals around relative predicted probabilities of step selection.

During spring, M5 indicated that female grizzly bear steps brought them closer to higher vegetation height seismic lines in non-forest, closer to lower vegetation height seismic lines in conifer forest, and further from higher vegetation height seismic lines in conifer forest (Fig 4). M3 indicated that during spring, regardless of seismic line wetness, male grizzly bear steps brought them closer to lower vegetation height seismic lines and further from higher vegetation height seismic lines (Fig 5). M5 indicated that during spring male grizzly bear steps brought them closer to seismic lines in non-forest and conifer forest, specifically closer to higher vegetation height seismic lines in non-forest, and closer to lower vegetation height seismic lines in conifer forest. M5 also indicated that, regardless of vegetation height, male grizzly bear steps brought them further from seismic lines in mixed forest (Fig 4).

**Fig 6:**
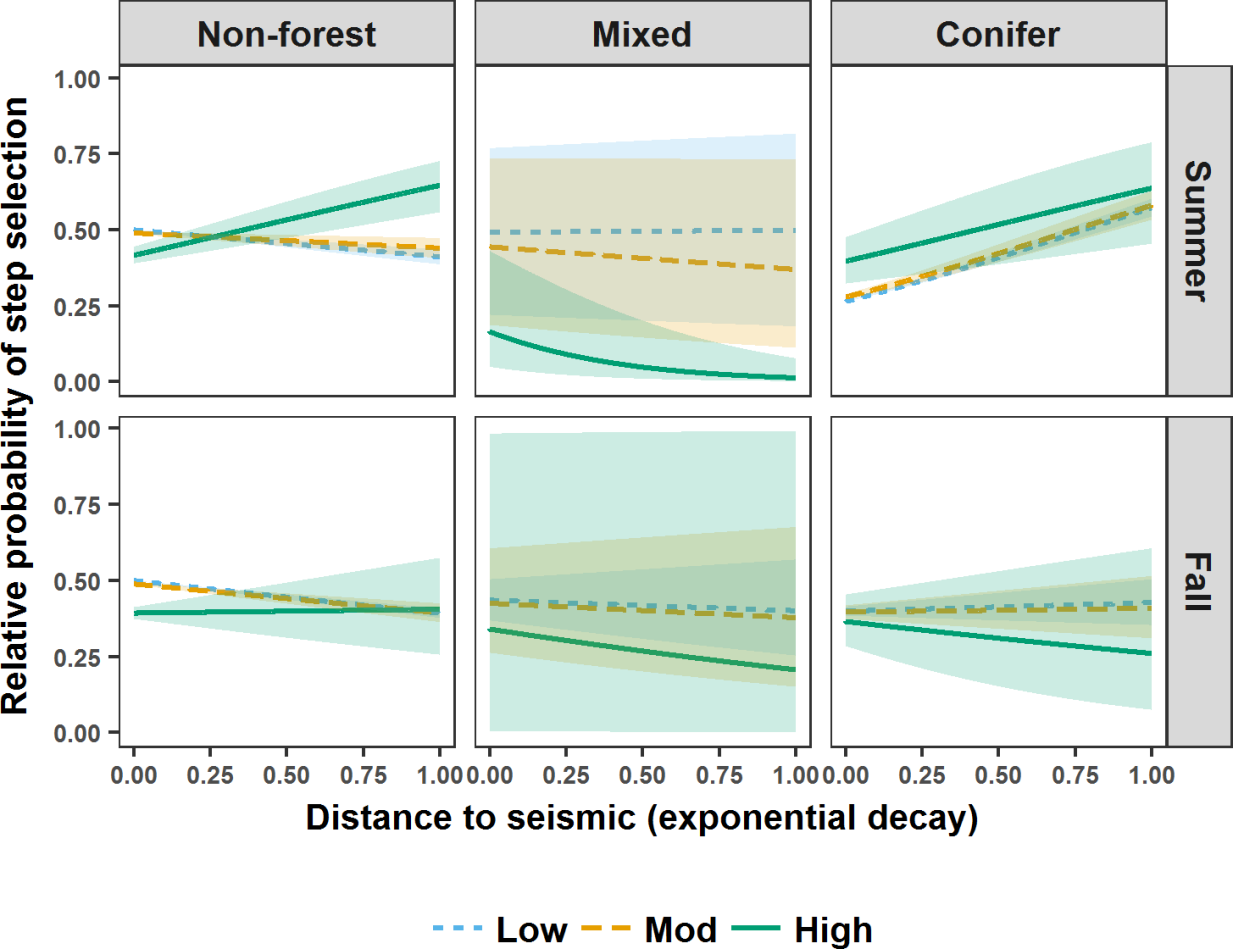
Relative probability of step selection by female grizzly bears in west-central Alberta during summer and fall. Relative probability of step selection by female grizzly bears in west-central Alberta between 2005 and 2009 during summer and fall in relation to distance to seismic lines (represented as an exponential decay (1-exp^-0.002* Distance (m)^)), seismic line vegetation height (visualised using the mean of the lower (Low), middle (Mod), and upper (High) quantiles), and landcover intersecting seismic lines (Non-forest, Mixed, Conifer). Shaded areas are 95% confidence intervals around relative predicted probabilities of step selection.

During summer, M5 indicated that female grizzly bear steps brought them closer to lower vegetation height seismic lines in non-forest, further from higher vegetation height seismic lines in non-forest, neither closer to nor further from seismic lines in mixed forest, and regardless of vegetation height, female grizzly bear steps brought them further from seismic lines in conifer forest (Fig 6). M1 indicated that during summer, male grizzly bear steps brought them further from seismic lines (β_*eDist*_ = 0.18, 95% CI = 0.14).

**Fig 7:**
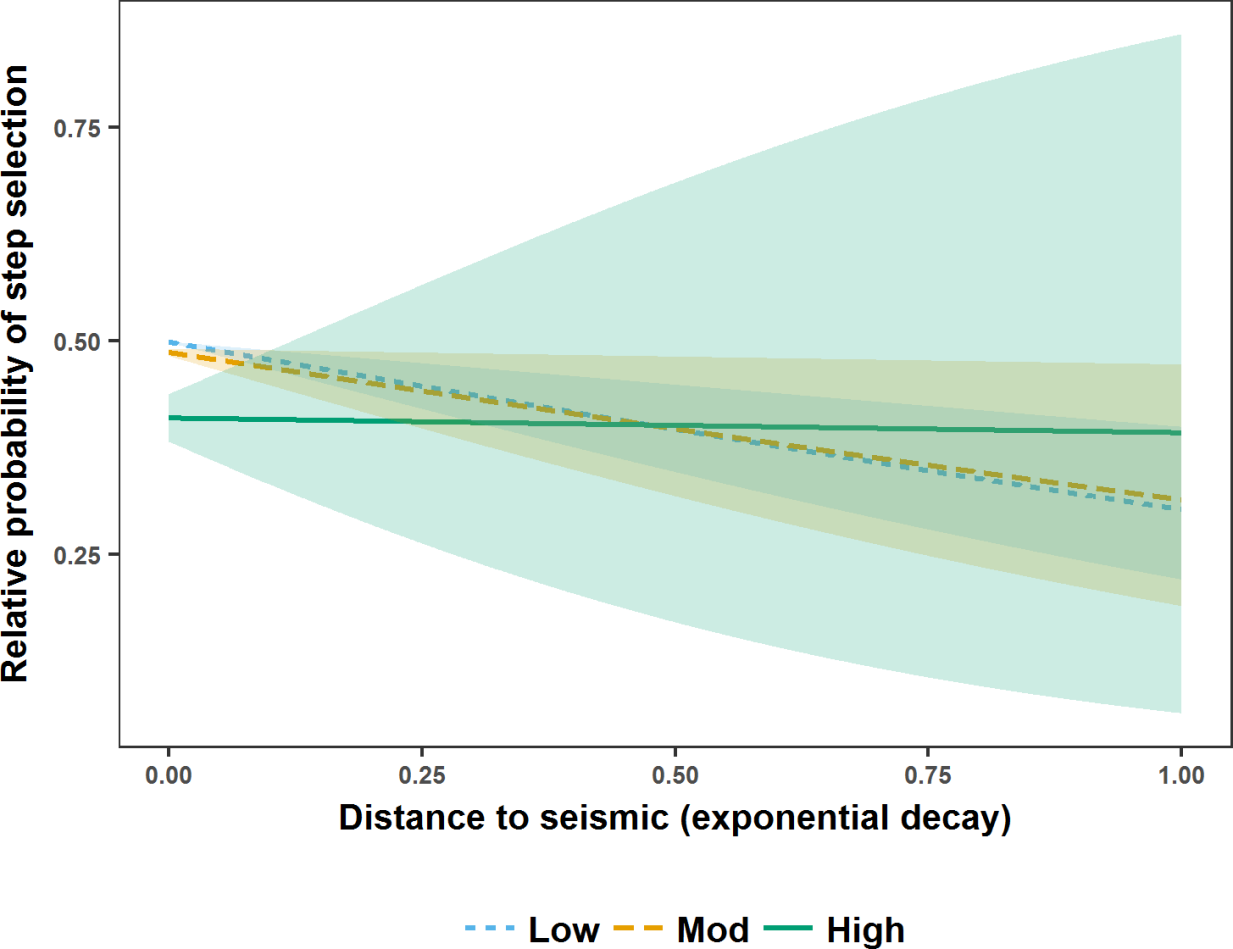
Relative probability of step selection by male grizzly bears in west-central Alberta during fall. Relative probability of step selection by male grizzly bears in west-central Alberta between 2005 and 2009 during fall in relation to distance to seismic lines (represented as an exponential decay (1-exp^-0.002* Distance (m)^)) and seismic line vegetation height (visualised using the mean of the lower (Low), middle (Mod), and upper (High) quantiles). Results are shown for the reference category of landcover intersecting seismic lines (Non-forest). Shaded areas are 95% confidence intervals around relative predicted probabilities of step selection.

During fall, M5 indicated that female grizzly bear steps brought them closer to lower vegetation height seismic lines in non-forest and regardless of vegetation height, neither closer to nor further from seismic lines in mixed and conifer forest (Fig 6). M5 also indicated that male grizzly bear steps brought them closer to lower vegetation height seismic lines regardless of landcover (Fig 7). Means and ranges of spearman rank correlations from k-fold cross validation suggested that all models failed to completely predict step selection of grizzly bears (Spring: Female mean r_s1_ 0.478, range -0.262–0.862; mean r_s0_−0.018, range -0.652–0.693, Male M3 mean r_s1_ 0.610, range 0.037–0.894; mean r_s0_−0.031, range -0.781–0.845, M5 mean r_s1_ 0.496, range -0.051–0.941; mean r_s0_−0.005, range -0.878–0.740; Summer: Female mean r_s1_ 0.483, range -0.027–0.902; r_s0_ 0.015, range -0.886–0.668, Male mean r_s1_ 0.-0.069, range -0.603–0.551; r_s0_−0.001, range -0.705–0.739; Fall: Female mean r_s_ 0.611, range 0.032–0.956, r_s_ 0.017, range -0.612–0.661, Male mean r_s1_ 0.529, range 0.018–0.945, r_s0_ 0.005, range -0.877–0.685).

### Movement rates–fine scale movements

#### Wolves

The best model explaining wolf movement rate included an interaction between vegetation height (*Veght*), season (f*Season*), and landcover (*fFor*; M8; Table A in S4 File), and random effect for individual (Table B in S4 File). M8 indicated that during the rendezvous season, wolf movement rate increased near lower vegetation height seismic lines in forest, but there was no relationship between wolf movement rate and vegetation height of seismic lines in non-forest, or between movement rate and vegetation height of seismic lines during other seasons (Table 2, R^2^_LMM(m)_ 0.06, R^2^_LMM(c)_ 0.07). *Post-hoc* piecewise linear regression comparing movement rate of wolves in the rendezvous season and vegetation height of seismic lines in forest revealed a decrease in wolf movement rate above vegetation heights of 0.7 m (95% CI = 0.06).

**Table 2:** Coefficient estimates (β) and 95% confidence intervals (CI) for the best model (M8) explaining female wolf and grizzly bear movement rate in west-central Alberta, Canada, between 2003 and 2009.

	Wolves		Female grizzly bears	
	β	±95% CI	β	±95% CI
log(Veght)	-0.386	1.110	-0.434	0.614
fSeason_(Rendezvous|Summer)_^1^	0.184	0.997	-0.317	0.247
fSeason_(Nomadic|Fall)_^2^	0.770	0.835	-0.114	0.255
fFor	-0.271	0.708	**0.347**	**0.321**
log(Veght)*fSeason_(Rendezvous|Summer)_^1^	0.697	2.207	0.179	0.691
log(Veght)*fSeason_(Nomadic|Fall)_^2^	-0.237	0.674	**0.742**	**0.363**
log(Veght)* fForest	2.109	1.111	0.151	0.707
fFor*fSeason_(Rendezvous|Summer)_^1^	**2.041**	**0.876**	-0.057	0.372
fFor*fSeason_(Nomadic|Fall)_^2^	-0.673	1.482	-0.303	0.373
log(Veght)*fFor*fSeason_(Rendezvous|Summer)_^1^	**-4.189**	**3.063**	-0.054	0.816
log(Veght)*fFor*fSeason_(Nomadic|Fall)_^2^	-1.695	2.331	-0.240	0.853

Significant relationships are shown in bold. Denning and Spring were the reference categories for fSeason for wolves, and grizzly bears respectively. Variables are described in S1 Table.

^**1**^ Results for wolves are for the Rendezvous season, and results for grizzly bears are for the Summer season.

^**2**^ Results for wolves are for the Nomadic season, and results for grizzly bears are for the Fall season.

#### Grizzly bears

The best model explaining female grizzly bear movement rate included an interaction between vegetation height (*Veght*), season (*fSeason*), and landcover (*fFor*; M8; Table A in S4 File). M8 indicated that during spring and summer, there was no relationship between female grizzly bear movement rate and vegetation height of the nearest seismic line. However, during fall, female grizzly bear movement rate increased near higher vegetation height seismic lines in non-forest (Table 2; R^2^_LMM(m)_ 0.02, R^2^_LMM(c)_ 0.04). The best model explaining male grizzly bear movement rate was the null model (M6; Table A in S4 File). M6 indicated that male grizzly bear movement rates were higher during spring (reference category) when compared to summer (β_Summer_ = -0.770, 95% CI = 0.393) and fall (β_Fall_ = -1.054, 95% CI = 0.381; R^2^_LMM(m)_ 0.09, R^2^_LMM(c)_ 0.14).

## Discussion

Unravelling the mechanisms driving wildlife response to regenerating anthropogenic disturbance is essential to understand the spatiotemporal effects of anthropogenic activity on wildlife, and could be used to direct habitat restoration efforts to benefit species of conservation concern. In Alberta, Canada, the primary focus of recovery efforts for threatened woodland caribou is restoration of habitat, and specifically, identifying linear features that should be prioritized for restoration within caribou ranges. We found stronger selection of seismic lines by wolves when compared to grizzly bears, but responses to seismic lines varied seasonally and were dependent on regeneration stage (i.e. vegetation height) and landcover. During the rendezvous season, wolves are likely using low vegetation height seismic lines to increase travel efficiency, but as wolves moved towards seismic lines regardless of vegetation height, our results suggest that seismic lines may not only be attractive to wolves as movement routes. For grizzly bears, movements in relation to seismic lines do not appear to increase travel efficiency. Combined, our results suggest that low vegetation height seismic lines primarily benefit wolves, facilitating movement, increasing wolf-ungulate encounters, and leading to a more rapid saturation of the wolf functional response [20].

As predicted, we found that wolves moved towards seismic lines with lower vegetation height during the rendezvous seasons. We also found that wolves moved faster near seismic lines with vegetation <0.7m, a result which is accordance with that of Dickie et al. [30], despite the comparatively coarse fix rates available for our analysis (2 hour vs. 5 min). Whether seismic lines with low vegetation are used to minimise the energetic costs of travel during the pup-rearing season [41, 79], or whether wolves move faster along low vegetation seismic lines because they are in areas with less ungulate prey, would require further investigation. Still, regardless of the mechanisms, seismic lines with lower vegetation heights likely provide movement routes for wolves during the rendezvous season, consistent with previous studies [25,26,74]. As prey are more diffuse and more difficult to encounter during the rendezvous season [41], the observed varations in movement behaviour among individual wolves are not suprising, and likely explain why we did not observe a cohesive response in movement behaviour relative to seismic lines during that season.

Seismic lines with lower vegetation may be attractive movement routes during snow-free months, however, when snow is on the ground during the nomadic season and part of the denning season, snowpack depth and compaction can hinder or benefit movement on seismic lines [79,80]. Snow depth and compaction data were unavailable for the ~15,000 km of seismic lines in this study but may explain why we found no relationship between wolf movement rates and vegetation height during the nomadic and denning seasons.

Contrary to our prediction, we found that, wolves moved towards higher vegetation seismic lines during the denning season, and regardless of vegetation height, wolves moved towards seismic lines during the nomadic season. One potential explanation is that despite the high horizontal resolution of LiDAR (1 m), we were unable to identify narrow game trails or even all-terrain vehicle (ATV) trails underneath broad tree canopies that could have been established at earlier stages of regeneration and maintained through continued animal use; Tigner et al. [28] reported game trails on seismic lines with high levels of regeneration. It also possible that altered understory communities caused by soil compaction during construction [81,82] make seismic lines attractive to wolves regardless of vegetation height, as they contain the combination of early seral stage vegetation and cover preferred by their ungulate prey [82–84]. The proposed link between wolf habitat selection, prey, and vegetation preferred by prey has been reported previously [85–87], and assessing moose, deer, and elk response to regenerating seismic lines in would help confirm our interpretations of wolf movement.

Movement of wolves towards seismic lines regardless of vegetation height during the denning and nomadic seasons is especially important because most wolf-caused mortality on caribou occurs during those seasons [88]. Our results suggest that with the goal of restoring ecosystem function for caribou, natural regeneration of seismic lines may be insufficient to successfully restore seismic lines [30], at least in the time-frames examined in this study. More active restoration activities such as tree planting or seismic line blocking (felling trees across seismic lines) could be required to reduce use of seismic lines by wolves. However, Neufeld [36] showed that even moderately large-scale seismic line restoration activities such as line blocking and tree falling failed to appreciably affect wolf selection for seismic lines. Thus, with respect to our question of when wolf use of seismic lines is indistinguishable from the surrounding landscape, our analyses suggest that there may not be a clear threshold at which this occurs, and that adaptive restoration treatments (e.g. line blocking, tree planting) combined with management of alternate prey [89] are likely required to reduce wolf selection for and continued use of seismic lines.

We found significant associations between grizzly bear selection and seismic lines that were in accordance with previous research on black bears (*U*. *americanus*) [28,29,90]. As predicted, we found that grizzly bears moved away from seismic lines with higher vegetation height, moved towards seismic lines with lower vegetation height during spring, and male grizzly bears also moved towards seismic lines with lower vegetation height during fall. However, contrary to our prediction we found no relationship between grizzly bear movement rates and vegetation height of seismic lines. As omnivores, movement of grizzly bears while feeding on vegetation or while hunting ungulates are difficult to separate from travelling or seeking mates, making interpretations more challenging. Therefore, it is possible that during spring grizzly bears are using seismic lines for travel, or to search for and pursue mates [91], and male grizzly bears may also be using seismic lines with lower vegetation height for travel during fall. However, it is also possible that grizzly bears are using lower vegetation height seismic lines not only as movement routes, but also to access herbaceous food and woody shrubs [82, 92–95]. Linking wildlife forage on seismic lines to LiDAR measurements of regeneration, combined with aforementioned assessments of moose, deer, and elk response to regenerating seismic lines, would confirm these potential explanations.

Within the exception of wolf models during the denning season, the poor cross validation results of suggest that seismic lines are not the only landscape feature influencing movements of wolves and grizzly bears within our study area. For grizzly bears, that forage in large areal disturbances such as forestry clear cuts and burned areas [7], and whose movements are driven by the availability of vegetative food [7], opportunistic hunting [96], and searching for mates [91], this result was not surprising. For wolves, although there is a link between seismic lines and movement [20,88], hunting behaviour is dependent on a range of additional factors such as prey density [97] and snow cover [80], which we were unable to include in our models. However, as ungulate specialists, wolf movement and habitat selection is driven by ungulate encounters [41, 87], therefore the strong cross validation of our denning models suggest that during that season seismic lines with higher vegetation height may be a source of ungulate prey. Habitat selection analysis of ungulate prey would confirm this interpretation.

## Conclusions

Based on our results and those of Dickie et al. [30], if restoration of linear features is soley focused on impeding wolf movement rate, then seismic lines with vegetation of less than 1 m could be prioritized for restoration. However, although regeneration did reduce grizzly bear response to seismic lines, because we found that wolves also moved towards seismic lines irrespective of vegetation height, in accordance with Dickie et al. [30], we caution the use of vegetation height alone as a metric to quantify habitat recovery. Instead, effective prioritization of habitat restoration, and ultimately, restoring ecosystem function for caribou will likely require a coordinated approach targeting disturbances preferred by alternate prey and shared-predators while considering the cumulative effects of disturbances across caribou ranges [98–100]. Ultimately, our results reveal that seismic lines continue to influence wolf movement behaviour decades after they are built, and even at later stages of regeneration, and it remains unknown at what stage of natural regeneration, if any, seismic lines cease to affect wolf movement. Considering that seismic lines may take up to 60 years to regenerate naturally in low productivity sites [32], it is likely that seismic lines are a long-term legacy within the boreal forest in western Canada. Avoiding new construction of these high-impact linear disturbance features elsewhere may help mitigate the long-term effects of anthropogenic activity on wildlife.

## Supporting information

S1 File Description of LiDAR processing used to attribute vegetation height to legacy seismic lines within the ranges of the Little Smoky, A La Peche, Redrock Prairie Creek and Narraway caribou herds in west-central Alberta, Canada. (DOCX)

S2 File Quasi-likelihood under the independence model criterion for candidate models used to identify factors determining broad scale movement behaviour of wolves and grizzly bears in west-central Alberta, Canada, between 2003 and 2009. (DOCX)

S3 File Population-level coefficient estimates and 95% confidence intervals for the best models explaining broad scale movement behaviour of wolves and grizzly bears in west-central Alberta, Canada, between 2003 and 2009. (DOCX)

S4 File Akaike under the independence model criterion for candidate models determining fine scale movement rates of wolves and grizzly bears in west-central Alberta, Canada, between 2003 and 2009. (DOCX)

S1 Table Variables used to explain broad scale movement behaviour (Step Selection Functions; SSF) and fine scale movement rates of wolves and grizzly bears in west-central Alberta, Canada, between 2003 and 2009. (DOCX)

S2 Table Number of individual wolves and grizzly bears (N), steps, clusters, and steps within 100 m of seismic lines used to explain broad scale movement behaviour (Step Selection Functions; SSF) and fine scale movement rates in west-central Alberta, Canada, between 2003 and 2009. Wolf clusters were successive steps taken < 5 days of one another while grizzly bear clusters were successive steps taken < 24 hours of one another. Wolf data were partitioned into denning, rendezvous, and nomadic seasons, while grizzly bear data were partitioned into males and females, and into spring, summer, and fall seasons.
(DOCX)

S1 Fig Histogram showing mean vegetation height (m) along 100 m segments of seismic lines in west-central Alberta, Canada, measured using LiDAR. (DOCX)

S2 Fig Graphical abstract to accompany Finnegan et al. Natural regeneration on seismic lines influences movement behaviour of wolves and grizzly bears. (TIFF)
