# Supplementary material for: Natural regeneration on seismic lines influences movement behaviour of wolves and grizzly bears
Source: PLoS One. 2018 Apr 16;13(4):e0195480. doi: 10.1371/journal.pone.0195480 (PMC5901995; doi:10.1371/journal.pone.0195480)
Supplement: S1 File — (DOCX) [file pone.0195480.s001.docx]

**S1 File. Description of LiDAR processing used to attribute vegetation height to legacy seismic lines within the ranges of the Little Smoky, A La Peche, Redrock Prairie Creek and Narraway caribou herds in west-central Alberta, Canada.**

The raw LiDAR signal returns (the Point Cloud) were partitioned into two sets of points: Bare Earth representing ground signals, and Full Feature representing returns from the forest canopy. These point datasets were then converted to ASCII text files of x, y, and z coordinates, and converted to Bare Earth (Digital Elevation model, or DEM, 1 m x 1 m resolution) and Full Feature (Digital Surface Model, or DSM, 1 m x 1 m resolution) grid surfaces at 1 m horizontal resolution. A canopy height surface was derived by subtracting the DEM from the DSM. We obtained seismic line features (polyline) from the Government of Alberta base features and used an automated geographic information systems (GIS) process to find paths with the lowest vegetation height (e.g. game trails) along these seismic lines. The GIS process clipped the canopy height surface to within 20 m of the original seismic line feature and generated a least-cost path raster (using vegetation height as the ‘cost’) between the start point and end point of the seismic line feature. The completed least-cost paths were converted to a line feature and divided into segments of approximately 100 m. Finally, the mean vegetation height for each 100 m segment was derived from the canopy height surface along the least-cost paths. We carried out GIS processing and vegetation height extractions in ArcGIS 10.2.2 [1].

# References

1. Environmental Systems Research Institute (ESRI). ArcGIS Desktop: Release 10. Redlands, California. Redlands, California; 2015.
