# Supplementary material for: Natural regeneration on seismic lines influences movement behaviour of wolves and grizzly bears
Source: PLoS One. 2018 Apr 16;13(4):e0195480. doi: 10.1371/journal.pone.0195480 (PMC5901995; doi:10.1371/journal.pone.0195480)
Supplement: S2 File — (DOCX) [file pone.0195480.s002.docx]

**S2 File. Quasi-likelihood under the independence model criterion for candidate models used to identify factors determining broad scale movement behaviour of wolves and grizzly bears in west-central Alberta, Canada, between 2003 and 2009.**

**Table A. Quasi-likelihood under the independence model criterion (QIC_U_), degrees of freedom (df), Log Likelihood, delta QIC_U_ (∆QIC_U_), and QIC_U_ weights (ω) for candidate models used to identify factors determining broad scale movement behaviour (Step Selection Functions) of wolves in west-central Alberta, Canada, between 2003 and 2009 during the denning, rendezvous and nomadic seasons.**

| **Season** | **Model** | **df** | **Log Likelihood** | **QIC** | **ΔQIC** | **ω** |
| --- | --- | --- | --- | --- | --- | --- |
| Denning | **5** | **11** | **-1545.747** | **3212.5** | **0** | **1** |
| (n = 3) | 2 | 3 | -1644.906 | 3319.2 | 106.76 | 0 |
|  | 3 | 7 | -1622.659 | 3335.5 | 122.99 | 0 |
|  | 4 | 7 | -1637.272 | 3337.7 | 125.26 | 0 |
|  | 1 | 1 | -1670.938 | 3363.2 | 150.76 | 0 |
| Rendezvous | **3** | **7** | **-2199.102** | **4430.3** | **0** | **0.851** |
| (n = 6) | 2 | 3 | -2207.419 | 4435.1 | 4.75 | 0.079 |
|  | 1 | 1 | -2215.225 | 4435.3 | 5.01 | 0.069 |
|  | 5 | 11 | -2190.321 | 4448.3 | 17.95 | 0 |
|  | 4 | 7 | -2201.599 | 4450.9 | 20.57 | 0 |
| Nomadic | **1** | **1** | **-11206.45** | **22424.4** | **0** | **0.995** |
| (n = 9) | 3 | 7 | -11165.21 | 22435 | 10.59 | 0.005 |
|  | 5 | 11 | -11151.21 | 22465.4 | 40.95 | 0 |
|  | 2 | 3 | -11203.07 | 22489.6 | 65.18 | 0 |
|  | 4 | 7 | -11194.81 | 22513.3 | 88.94 | 0 |

Sample sizes are in parenthesis. Best models are in bold. Model numbers correspond to those described, along with corresponding hypotheses, in Table 1.

**Table B. Quasi-likelihood under the independence model criterion (QIC_U_), degrees of freedom (df), Log Likelihood, delta QIC_U_ (∆QIC_U_), and QIC_U_ weights (ω) for candidate models used to identify factors determining broad scale movement behaviour (Step Selection Functions) of female grizzly bears in west-central Alberta, Canada, between 2005 and 2009 .**

| **Season** | **Model** | **df** | **Log Likelihood** | **QIC** | **ΔQIC** | **ω** |
| --- | --- | --- | --- | --- | --- | --- |
| Spring | **5** | **11** | **-4295.026** | **8617.9** | **0** | **1** |
| (n = 6) | 1 | 1 | -4323.466 | 8650.1 | 32.16 | 0 |
|  | 2 | 3 | -4321.498 | 8656.1 | 38.18 | 0 |
|  | 4 | 7 | -4317.527 | 8659.2 | 41.34 | 0 |
|  | 3 | 7 | -4320.984 | 8662.3 | 44.43 | 0 |
| Summer | **5** | **11** | **-7510.232** | **15052.6** | **0** | **1** |
| (n = 8) | 4 | 7 | -7555.203 | 15126.9 | 74.33 | 0 |
|  | 2 | 3 | -7562.682 | 15134.7 | 82.1 | 0 |
|  | 3 | 7 | -7560.976 | 15146.6 | 94.02 | 0 |
|  | 1 | 1 | -7573.861 | 15155.2 | 102.56 | 0 |
| Fall | **5** | **11** | **-9026.677** | **18081.1** | **0** | **1** |
| (n = 7) | 3 | 7 | -9039.91 | 18108 | 26.83 | 0 |
|  | 2 | 3 | -9050.733 | 18114 | 32.83 | 0 |
|  | 4 | 7 | -9044.948 | 18116.1 | 34.98 | 0 |
|  | 1 | 1 | -9061.447 | 18131.2 | 50.07 | 0 |

Sample sizes are in parenthesis. Best models are in bold. Model numbers correspond to those described, along with corresponding hypotheses, in Table 1.

**Table C. Quasi-likelihood under the independence model criterion (QIC_U_), degrees of freedom (df), Log Likelihood, delta QIC_U_ (∆QIC_U_), and QIC_U_ weights (ω) for candidate models used to identify factors determining broad scale movement behaviour (Step Selection Functions) of male grizzly bears in west-central Alberta, Canada, between 2005 and 2009.**

| **Season** | **Model** | **df** | **Log Likelihood** | **QIC** | **ΔQIC** | **ω** |
| --- | --- | --- | --- | --- | --- | --- |
| Spring | **3** | **7** | **-1680.469** | **3380.1** | **0** | **0.648** |
| (n = 3) | **5** | **11** | **-1675.823** | **3381.5** | **1.33** | **0.333** |
|  | 2 | 3 | -1687.077 | 3387.2 | 7.03 | 0.019 |
|  | 4 | 7 | -1679.977 | 3400.1 | 19.92 | 0 |
|  | 1 | 1 | -1699.737 | 3404.9 | 24.79 | 0 |
| Summer | **1** | **1** | **-3121.927** | **6245.9** | **0** | **0.68** |
| (n = 4) | 4 | 7 | -3116.338 | 6248.4 | 2.53 | 0.192 |
|  | 2 | 3 | -3120.135 | 6250.2 | 4.28 | 0.08 |
|  | 3 | 7 | -3117.533 | 6252.5 | 6.63 | 0.025 |
|  | 5 | 11 | -3111.713 | 6252.6 | 6.74 | 0.023 |
| Fall | **5** | **11** | **-5156.442** | **10364.4** | **0** | **1** |
| (n = 4) | 3 | 7 | -5173.061 | 10380.5 | 16.06 | 0 |
|  | 2 | 3 | -5180.927 | 10385 | 20.54 | 0 |
|  | 1 | 1 | -5193.682 | 10390.9 | 26.44 | 0 |
|  | 4 | 7 | -5175.53 | 10400.6 | 36.21 | 0 |

Sample sizes are in parenthesis. Best models are in bold. Model numbers correspond to those described, along with corresponding hypotheses, in Table 1.
