# Supplementary material for: Natural regeneration on seismic lines influences movement behaviour of wolves and grizzly bears
Source: PLoS One. 2018 Apr 16;13(4):e0195480. doi: 10.1371/journal.pone.0195480 (PMC5901995; doi:10.1371/journal.pone.0195480)
Supplement: S3 File — (DOCX) [file pone.0195480.s003.docx]

S3 File. Population-level coefficient estimates and 95% confidence intervals for the best models explaining broad scale movement behaviour of wolves and grizzly bears in west-central Alberta, Canada, between 2003 and 2009.

Table A. Population-level coefficient estimates (β) and 95% confidence intervals (CI) for the best models (M1, M3, M5) explaining broad scale movement behaviour (Step Selection Functions) of wolves in west-central Alberta, Canada, between 2003 and 2009.

|  | **Denning (n = 3): M5** | | | **Rendezvous (n = 6): M3** | | | **Nomadic (n = 9): M1** | | |
| --- | --- | --- | --- | --- | --- | --- | --- | --- | --- |
| **Variables** | **β** | **± 95% CI** | **+\|-** | **β** | **± 95% CI** | **+\|-** | **β** | **± 95% CI** | **+\|-** |
| eDist | **-3.095** | **0.087** | 0\|2 | **-0.753** | **0.062** | 0\|3 | **-0.330** | **0.013** | 3\|3 |
| Veght | **-0.726** | **0.048** | 3\|0 | **-0.212** | **0.002** | 1\|2 | **-** | **-** | **-** |
| eWAM | **-** | **-** | **-** | **-0.897** | **0.082** | 1\|2 | **-** | **-** | **-** |
| fLand(Con) | **-1.772** | **0.059** | 0\|2 | **-** | **-** |  | **-** | **-** | **-** |
| fLand(Mix) | -0.298 | 0.345 | 1\|2 | **-** | **-** |  | **-** | **-** | **-** |
| eDist*Veght | **1.204** | **0.078** | 3\|0 | **0.411** | **0.073** | 1\|0 | **-** | **-** | **-** |
| eDist*eWAM | **-** | **-** | **-** | 0.235 | 1.650 | 1\|1 | **-** | **-** | **-** |
| Veght*eWAM | **-** | **-** | **-** | **0.246** | **0.005** | 2\|1 | **-** | **-** | **-** |
| eDist*fLand(Con) | **2.599** | **0.213** | 2\|0 | **-** | **-** | **-** | **-** | **-** | **-** |
| eDist*fLand(Mix) | **3.359** | **0.072** | 3\|0 | **-** | **-** | **-** | **-** | **-** | **-** |
| Veght*fLand(Con) | **1.041** | **0.053** | 3\|0 | **-** | **-** | **-** | **-** | **-** | **-** |
| Veght*fLand(Mix) | **0.635** | **0.194** | 2\|0 | **-** | **-** | **-** | **-** | **-** | **-** |
| eDist*Veght*eWAM | **-** | **-** | **-** | -0.096 | 0.474 | 0\|0 | **-** | **-** | **-** |
| eDist*Veght* fLand(Con) | **-2.079** | **0.092** | 0\|3 | **-** | **-** | **-** | **-** | **-** | **-** |
| eDist*Veght* fLand(Mix) | **-4.031** | **0.153** | 0\|3 | **-** | **-** | **-** | **-** | **-** | **-** |

Significant relationships are shown in bold. The number of individuals with significant positive (+) and negative (-) coefficients (95% CI not overlapping zero) are also shown. The reference category for fLand was non-forest (fLand: NF). Variables, models (M), and associated predictions are described in Table 1 and S1 Table.

**Table B***.* **Coefficient estimates** (**β**) **and 95% confidence intervals (CI) for the best model (M5) explaining broad scale movement behaviour (Step Selection Functions) of female grizzly bears in west-central Alberta, Canada, between 2005 and 2009.**

|  | **Spring (n = 6)** | | | **Summer (n = 8)** | | | **Fall (n = 7)** | | |
| --- | --- | --- | --- | --- | --- | --- | --- | --- | --- |
| **Variables** | **β** | **± 95% CI** | **+\|-** | **β** | **± 95% CI** | **+\|-** | **β** | **± 95% CI** | **+\|-** |
| eDist | **-0.058** | **1.587** | 0\|0 | **-0.373** | **0.104** | 0\|2 | **-0.436** | **0.061** | 0\|2 |
| Veght | **0.393** | **0.003** | 0\|0 | **-0.147** | **0.055** | 0\|1 | **-0.277** | **0.054** | 1\|1 |
| fLand(Con) | **-0.339** | **0.009** | 0\|1 | **-1.037** | **0.004** | 0\|2 | **-0.412** | **0.062** | 0\|3 |
| fLand(Mix) | -0.152 | 0.550 | 0\|0 | -0.026 | 1.233 | 2\|1 | **-0.268** | **0.246** | 0\|1 |
| eDist*Veght | **-0.501** | **0.070** | 0\|2 | **0.576** | **0.069** | 1\|1 | **0.444** | **0.226** | 1\|0 |
| eDist*fLand(Con) | **-0.741** | **0.061** | 0\|1 | **1.698** | **0.015** | 1\|0 | **0.567** | **0.183** | 1\|0 |
| eDist*fLand(Mix) | 0.179 | 2.025 | 0\|0 | **0.417** | **0.162** | 1\|3 | 0.303 | 0.334 | 1\|1 |
| Veght*fLand(Con) | **-0.903** | **0.001** | 0\|1 | 0.121 | 0.086 | 1\|1 | **0.182** | **0.138** | 1\|0 |
| Veght*fLand(Mix) | **0.064** | **0.061** | 1\|1 | **-0.866** | **0.001** | 1\|2 | 0.015 | 2.710 | 1\|0 |
| eDist*Veght* fLand(Con) | **1.135** | **0.017** | 2\|0 | **-0.736** | **0.074** | 3\|1 | **-0.688** | **0.205** | 0\|2 |
| eDist*Veght* fLand(Mix) | **-0.324** | **0.076** | 1\|1 | **-1.811** | **0.047** | 4\|1 | **-0.645** | **0.272** | 0\|0 |

Significant relationships are shown in bold. The number of individuals with significant positive (+) and negative (-) coefficients (95% CI not overlapping zero) are also shown. The reference category for fLand was non-forest (fLand: NF). Variables, models (M4), and associated predictions are described in Table 1 and S1 Table.

**Table C***.* **Coefficient estimates** (**β**) **and 95% confidence intervals (CI) for the best models (M1, M3, M5) explaining broad scale movement behaviour (Step Selection Functions) of male grizzly bears in west-central Alberta, Canada, between 2005 and 2009.**

|  | **Spring (n = 4*)*** | | | | | | **Summer (n = 4)** | | | **Fall (n = 4)** | | |
| --- | --- | --- | --- | --- | --- | --- | --- | --- | --- | --- | --- | --- |
|  | **M3** | | |  | **M5** |  | **M1** | | | **M5** | | |
| **Variables** | **β** | **± 95% CI** | **+\|-** | **β** | **± 95% CI** | **+\|-** | **β** | **± 95% CI** | **+\|-** | **β** | **± 95% CI** | **+\|-** |
| eDist | **-1.354** | **0.001** | 0\|2 | **-0.813** | **0.015** | **0\|4** | **0.184** | **0.135** | 1\|0 | **-0.849** | **0.039** | 0\|2 |
| Veght | **-1.740** | **0.018** | 0\|1 | **0.760** | **0.008** | 2\|0 | **-** | **-** | **-** | **-0.194** | **0.059** | 0\|1 |
| eWAM | **0.355** | **0.012** | 1\|0 | **-** | **-** | **-** | **-** | **-** | **-** | **-** | **-** | **-** |
| fLand(Con) | **-** | **-** | **-** | **0.127** | **0.104** | 0\|0 | **-** | **-** | **-** | **-0.989** | **0.028** | 0\|2 |
| fLand(Mix) | **-** | **-** | **-** | **-0.293** | **0.305** | 0\|0 | **-** | **-** | **-** | 0.036 | 2.795 | 1\|1 |
| eDist*Veght | **4.317** | **0.093** | 1\|0 | **-0.868** | **0.001** | 1\|2 | **-** | **-** | **-** | **0.404** | **0.098** | 1\|0 |
| eDist*eWAM | **0.843** | **0.034** | 2\|0 | **-** | **-** | **-** | **-** | **-** | **-** | **-** | **-** | **-** |
| Veght*eWAM | **1.496** | **0.027** | 1\|0 | **-** | **-** | **-** | **-** | **-** | **-** | **-** | **-** | **-** |
| eDist*fLand(Con) | **-** | **-** | **-** | -0.072 | 0.091 | 0\|0 | **-** | **-** | **-** | **0.599** | **0.089** | 2\|0 |
| eDist*fLand(Mix) | **-** | **-** | **-** | **1.176** | **0.396** | 1\|1 | **-** | **-** | **-** | **0.786** | **0.494** | 0\|1 |
| Veght*fLand(Con) | **-** | **-** | **-** | **-0.579** | **0.018** | 0\|3 | **-** | **-** | **-** | 0.107 | 0.112 | 1\|1 |
| Veght*fLand(Mix) | **-** | **-** | - | **-0.899** | **0.001** | 0\|2 | **-** | **-** | **-** | -0.010 | 4.466 | 0\|0 |
| eDist*Veght*eWAM | **-3.071** | **0.193** | 0\|1 | **-** | **-** | **-** | **-** | **-** | **-** | **-** | **-** | **-** |
| eDist*Veght* fLand(Con) | **-** | **-** | **-** | **0.563** | **0.011** | 2\|0 | **-** | **-** | **-** | -0.190 | 0.246 | 1\|1 |
| eDist*Veght* fLand(Mix) | **-** | **-** | **-** | **1.049** | **0.017** | 2\|0 | **-** | **-** | **-** | -0.062 | 1.690 | 0\|0 |

Significant relationships are shown in bold. The number of individuals with significant positive (+) and negative (-) coefficients (95 % CI not overlapping zero) are also shown. The reference category for fLand was non-forest (fLand: NF). Variables, models (M), and associated predictions are described in Table 1 and S1 Table.
