# Supplementary material for: Natural regeneration on seismic lines influences movement behaviour of wolves and grizzly bears
Source: PLoS One. 2018 Apr 16;13(4):e0195480. doi: 10.1371/journal.pone.0195480 (PMC5901995; doi:10.1371/journal.pone.0195480)
Supplement: S4 File — (DOCX) [file pone.0195480.s004.docx]

**S4 File. Akaike under the independence model criterion for candidate models determining fine scale movement rates of wolves and grizzly bears in west-central Alberta, Canada, between 2003 and 2009.**

**Table A. Akaike under the independence model criterion (AIC_c_), degrees of freedom (df), Log Likelihood, delta AIC_c_ (∆AIC_c_), and AIC_c_ weights (ω) for candidate models (M) used to identify factors determining fine scale movement rate of wolves and grizzly bears in west-central Alberta, Canada, between 2003 and 2009.**

|  | **Model** | **df** | **Log Likelihood** | **AIC_C_** | **ΔAIC_C_** | **ω** |
| --- | --- | --- | --- | --- | --- | --- |
| Wolves | **M8** | **14** | **-1839.21** | **3706.94** | **0** | **1** |
|  | M7 | 8 | -1854.58 | 3725.33 | 18.39 | 0 |
|  | M6 | 5 | -1859.96 | 3730 | 23.06 | 0 |
| Female grizzly bears | **M8** | **14** | **-3118.5** | **6265.22** | **0** | **1** |
|  | M7 | 8 | -3295.8 | 6607.67 | 342.45 | 0 |
|  | M6 | 5 | -3299.75 | 6609.53 | 344.31 | 0 |
| Male grizzly bears | **M6** | **5** | **-1194** | **2398.08** | **0** | **0.51** |
|  | M7 | 8 | -1191.26 | 2398.72 | 0.64 | 0.37 |
|  | M8 | 14 | -1186.27 | 2401.11 | 3.04 | 0.11 |

M6 was the null model. The best model is in bold. Models (M) correspond to those described, along with corresponding predictions, in Table 1.

**Table B. Akaike under the independence model criterion (AIC_c_), degrees of freedom (df), Log Likelihood, delta AIC_c_ (∆AIC_c_) and AIC_c_ weights (ω) for candidate models comparing the random effect structure of the best model (M8) explaining wolf movement rate in west-central Alberta, Canada, between 2003 and 2009.**

| **Random effect structure** | **df** | **Log Likelihood** | **AIC_C_** | **ΔAIC_C_** | **ω** |
| --- | --- | --- | --- | --- | --- |
| 1\|Individual | **14** | **-1839.21** | **3706.94** | **0** | **0.68** |
| 1\|Pack/Individual | 15 | -1838.92 | 3708.45 | 1.51 | 0.32 |

The best model describing the random effect structure is in bold.
