# Supplementary material for: Natural regeneration on seismic lines influences movement behaviour of wolves and grizzly bears
Source: PLoS One. 2018 Apr 16;13(4):e0195480. doi: 10.1371/journal.pone.0195480 (PMC5901995; doi:10.1371/journal.pone.0195480)
Supplement: S1 Table — (DOCX) [file pone.0195480.s005.docx]

**S1 Table. Variables used to explain broad scale movement behaviour (Step Selection Functions; SSF) and fine scale movement rates of wolves and grizzly bears in west-central Alberta, Canada, between 2003 and 2009.**

| **Variable** | **Description** | **Variable Type** | **Range** | **Broad Scale: SSF** | **Fine Scale: Movement Rate** |
| --- | --- | --- | --- | --- | --- |
| Veght^1^ | Mean height of vegetation along 100 m section of seismic line, m. | Continuous | 0 – 15 | x | x |
| eWAM | Mean wet areas mapping (depth to water, m) underneath seismic line sections represented as an exponential decay function with a threshold of 3 m. | Continuous | 0 – 1 | x |  |
| eDist | Distance to the nearest seismic line (m) represented as an exponential decay function with a threshold of 500 m. | Continuous | 0 – 1 | x |  |
| Density | Mean density of seismic lines intersecting seismic line sections, km/km^2^. | Continuous | 0 – 9 | x |  |
| fLand | Landcover intersecting with the majority of the seismic line section: Non-forest (NF) ≤ 5% trees, Mixed (Mix) > 5% trees, < 80% conifer, Conifer (Con) > 5% trees, ≥ 80% conifer. | Factor | NA | x |  |
| fFor | Landcover intersecting with the majority of the seismic line section: Non-Forest (0) ≤ 5% trees, Forest (1) > 5% trees. | Binary | 0/1 |  | x |

^1^ For movement rate analysis we log_e_ transformed *Veght* after adding a constant of 1.
