# Supplementary material for: Natural regeneration on seismic lines influences movement behaviour of wolves and grizzly bears
Source: PLoS One. 2018 Apr 16;13(4):e0195480. doi: 10.1371/journal.pone.0195480 (PMC5901995; doi:10.1371/journal.pone.0195480)
Supplement: S2 Table — Wolf clusters were successive steps taken < 5 days of one another while grizzly bear clusters were successive steps taken < 24 hours of one another. Wolf data were partitioned into denning, rendezvous, and nomadic seasons, while grizzly bear data were partitioned into males and females, and into spring, summer, and fall seasons. (DOCX) [file pone.0195480.s006.docx]

**S2 Table. Number of individual wolves and grizzly bears (N), steps, clusters, and steps within 100 m of seismic lines used to explain broad scale movement behaviour (Step Selection Functions; SSF) and fine scale movement rates in west-central Alberta, Canada, between 2003 and 2009.**

|  | **Broad scale: SSF** | | | **Fine scale: Movement rate (m/hr)** | | **% locations <100m from seismic line** |
| --- | --- | --- | --- | --- | --- | --- |
| **Season** | **N** | **Steps** | **Clusters** | **N** | **Steps** |  |
| **Wolves** |  |  |  |  |  |  |
| Denning | 3 | 590 | 9 | 2 | 82 | 14 |
| Rendezvous | 6 | 710 | 26 | 3 | 123 | 17 |
| Nomadic | 9 | 3,367 | 32 | 6 | 608 | 18 |
| **Grizzly bear males** |  |  |  |  |  |  |
| Spring | 3 | 510 | 8 | 3 | 148 | 29 |
| Summer | 4 | 925 | 31 | 3 | 234 | 25 |
| Fall | 4 | 1,532 | 34 | 3 | 366 | 24 |
| **Grizzly bear females** |  |  |  |  |  |  |
| Spring | 6 | 1,357 | 50 | 3 | 281 | 21 |
| Summer | 8 | 2,251 | 74 | 6 | 854 | 38 |
| Fall | 7 | 3,083 | 110 | 6 | 903 | 29 |

Wolf clusters were successive steps taken < 5 days of one another while grizzly bear clusters were successive steps taken < 24 hours of one another. Wolf data were partitioned into denning, rendezvous, and nomadic seasons, while grizzly bear data were partitioned into males and females, and spring, summer, and fall seasons.
