# Supplementary material for: Natural regeneration on seismic lines influences movement behaviour of wolves and grizzly bears
Source: PLoS One. 2018 Apr 16;13(4):e0195480. doi: 10.1371/journal.pone.0195480 (PMC5901995; doi:10.1371/journal.pone.0195480)

**S1 Fig. Histogram showing mean vegetation height (m) along 100 m segments of seismic lines in west-central Alberta, Canada, measured using LiDAR.**


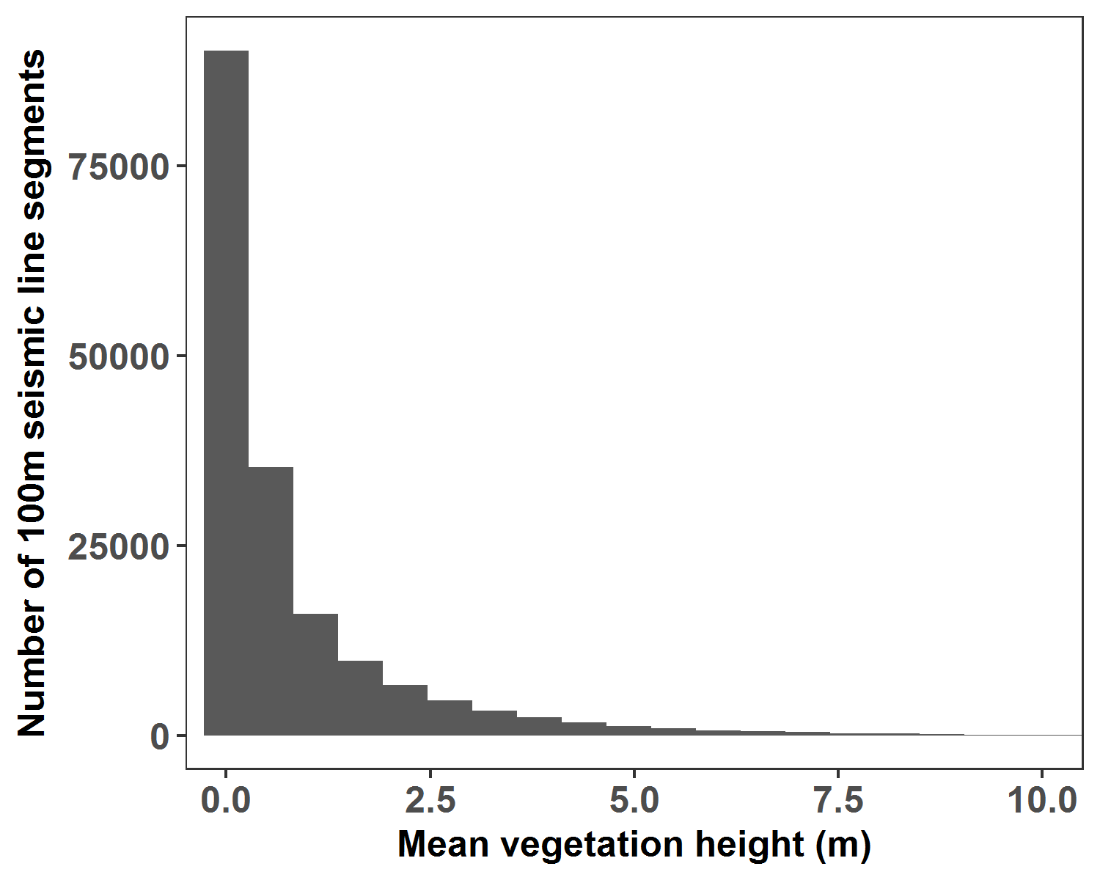

Supplement: S1 Fig — (DOCX) [file pone.0195480.s007.docx]
