# Supplementary figures and images for: Natural regeneration on seismic lines influences movement behaviour of wolves and grizzly bears
Source: PLoS One. 2018 Apr 16;13(4):e0195480. doi: 10.1371/journal.pone.0195480 (PMC5901995; doi:10.1371/journal.pone.0195480)

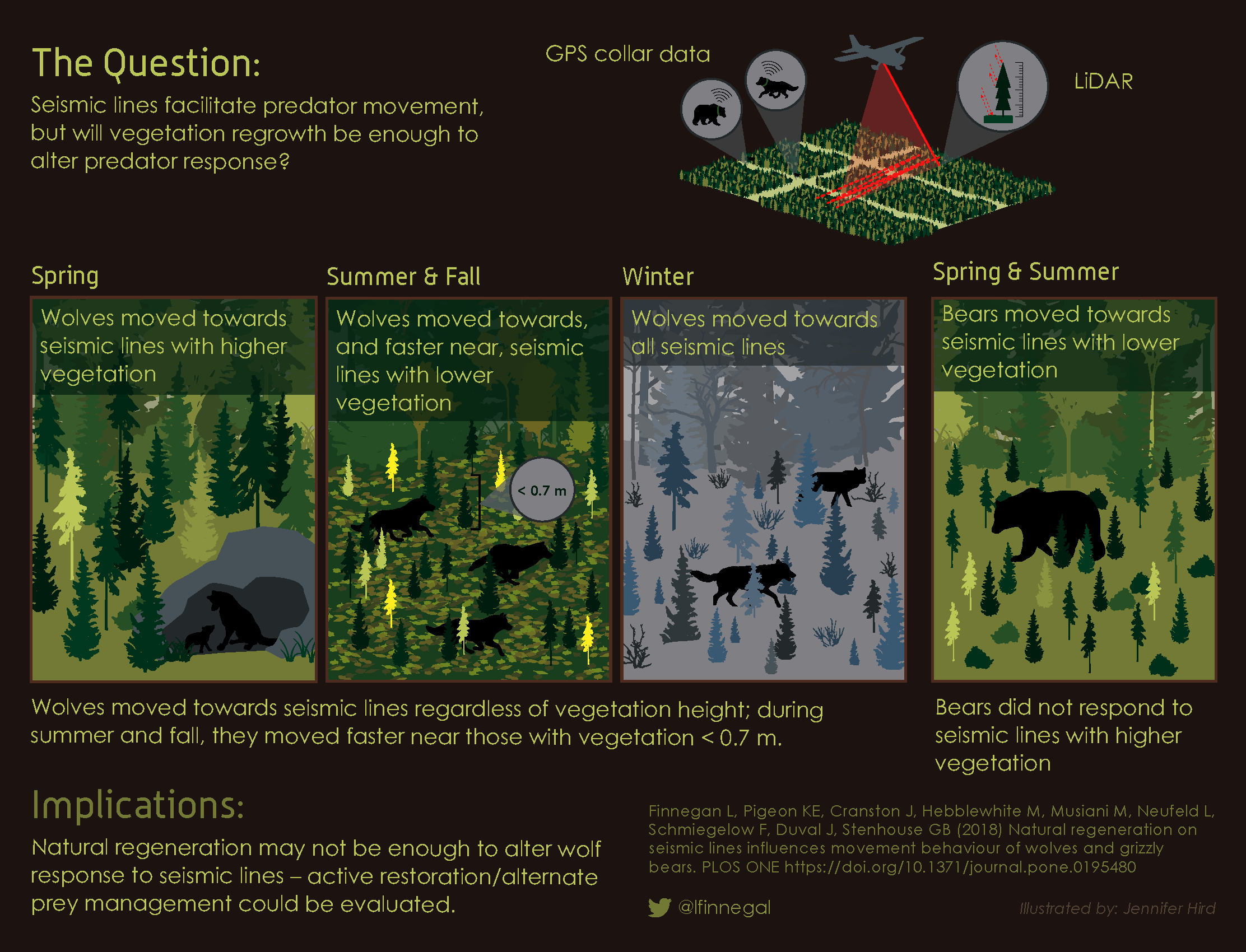

Supplement: S2 Fig — (TIFF) [file pone.0195480.s008.tiff]
